# Impact of Age on Success of 3D-Printed Cranial Remolding Orthoses for Deformational Head Shapes

**DOI:** 10.3390/jcm15187002

**Published:** 2026-09-10

**Authors:** Emilia Cotter, Maria Araujo, Alyssa Juma, Jijia Wang, Tiffany Graham

**Affiliations:** 1 ROKband Pediatric Headshape Clinics, Unit 801-625 Fifth Avenue, New Westminster, BC V3M 1X4, Canada; emilia@rokbandclinics.ca (E.C.);; 2 Department of Applied Clinical Research, School of Health Professions, University of Texas Southwestern Medical Center, 6011 Harry Hines Blvd, Dallas, TX 75390-9091, USA; jijia.wang@utsouthwestern.edu; 3 Department of Prosthetics-Orthotics, School of Health Professions, University of Texas Southwestern Medical Center, 6011 Harry Hines Blvd, Dallas, TX 75390-9091, USA

**Keywords:** cranial remolding orthosis, deformational plagiocephaly, brachycephaly, 3D printing, cranial asymmetry, infant, helmet therapy, cranial deformation

## Abstract

**Background/Objectives**: Previous studies regarding the efficacy of cranial remolding orthoses (CROs) have shown that earlier treatment initiation is associated with more favorable outcomes, but there are few studies utilizing 3D-printed CROs. This study evaluated the impact of treatment initiation age on the success of 3D-printed CROs in terms of morphological measurement changes and caregiver satisfaction. **Methods**: A total of 1054 infants who initiated treatment with a 3D-printed CRO during 2023 across six pediatric clinics in Canada were included in this retrospective cohort study. Participants were stratified by treatment initiation age (<6 months, 6–9 months, and 9–12 months) and deformational head shape: deformational plagiocephaly (DP), deformational brachycephaly (DB), or deformational asymmetric brachycephaly (DAB). **Results**: Relevant pre-treatment measurements for DP were CVAI = 7.98 ± 2.30, for DB were CR = 97 ± 4%, and DAB were CVAI = 7.22 ± 2.21 and CR = 95 ± 3%. All head shapes improved during treatment and overall caregiver satisfaction was high (94.4%). A total of 66.89% of participants achieved full correction of their cranial deformation (CR ≤ 90% and CVAI < 3.5), with the highest correction observed among the youngest infants for each head shape type. Cranial correction rates were highest for DP (76.85%), followed by DAB (57.73%) and DB (54.68%). The mean correction rate varied greatly between treatment initiation age and deformation type subgroups (21.74% to 82.73%). **Conclusions**: Treatment with 3D-printed CROs resulted in measurement improvement across all participants and caregiver satisfaction remained high. Earlier treatment initiation was associated with significantly better correction rates, supporting early referral and intervention whenever possible. These findings provide evidence to inform clinical recommendations and support caregivers in making decisions regarding treatment for cranial deformation and appear akin to the findings of studies utilizing traditionally fabricated CROs.

## 1. Introduction

The prevalence of infant head shape deformation has increased significantly over the last few decades as a result of the introduction of the “Back to Sleep” program in the 1990’s [1,2,3,4]. Previous findings suggest 20–50% of infants have cranial deformation [5,6,7]. While conservative therapy (such as active repositioning) may correct head shapes at young ages [8], the use of traditionally-fabricated Cranial Remolding Orthoses (CROs) have been shown to produce more desirable post-treatment measurements when compared to these more conservative options [9,10,11,12].

Cranial deformations exist due to an outside force acting on the infant skull, and deformational head shape types are named based on their visual presentation [6]: deformational plagiocephaly (DP), deformational brachycephaly (DB), and deformational asymmetric brachycephaly (DAB) are the most commonly reported head shapes [9,10]. In DP, one side of the posterior aspect of the head is flatter than the other causing an asymmetric head shape. DB is characterized by a central posterior head flattening, producing a wider than normal head shape [10]. A combination of DP and DB may also be present (DAB).

Risk factors for cranial asymmetry include the presence of torticollis (a congenital muscular condition affecting the neck), positional preferences, and factors related to pregnancy [13,14]. Limited research suggests that untreated DP can lead to cognitive and developmental delays in moderate to severe cases [15,16]. Untreated DP may negatively impact quality of life or increase psychosocial issues [17], facial asymmetry [18,19], and ear asymmetry [20,21]. The effectiveness of CRO treatment has been investigated over the last few decades [9,10,22,23,24]. There is a general consensus that the primary factors influencing CRO treatment include initial severity, treatment starting age, and CRO wear-time compliance [25,26,27,28]. Current research primarily focuses on the use of traditional CROs (made of plastic with a thick foam lining), with little investigation into the effectiveness of newer 3D-printed CROs [11,20,22,29,30,31].

The standard clinical procedures surrounding the treatment of deformational head shapes with a CRO involve not only the custom fabrication of the helmet, which uniquely fits the infant [3,24,29,31], but also include regular adjustments that should be performed by a trained practitioner every few weeks throughout treatment while the infant is actively growing [3,12]. The CRO shape depends on the infant’s presenting cranial shape, which is used as a starting template to create a more desired cranial shape. Through the use of custom software, the manufacturer creates the CRO with void spaces where the cranium is flattened and touches the areas where the infant’s skull is bossed. This final CRO shape varies based on the infant’s presentation at the initiation of orthotic treatment [11,22,25,26,28] and the desired post-treatment measurements [22,24,29,31]. Treatment duration can vary based on the amount of cranial correction needed and the infant’s individual growth rate; studies surrounding traditionally fabricated CROs generally report treatment periods of a few months, and a 2023 literature review recommended the CRO be worn at least 20 h per day for a minimum of 75 days to achieve meaningful cranial measurement improvements [23].

Studies evaluating caregiver satisfaction with treatment using traditionally fabricated CROs is frequently favorable [32,33,34,35]; however, there are currently limited studies that look at the satisfaction from caregivers when treating the cranial deformation with 3D printed CROs [36], and there is a lack of research surrounding the impact of treatment initiation age on caregiver satisfaction in this population.

This study evaluates the likelihood of achieving normalization of cranial measurements and post-treatment caregiver satisfaction among infants treated with 3D-printed CROs, stratified by treatment initiation age. This analysis will aid clinicians and caregivers in making informed decisions regarding the ideal age of initiation of 3D-printed CRO treatment.

## 2. Materials and Methods

This retrospective cohort study examined treatment outcomes for patients who were treated with custom-fabricated 3D-printed CROs at six Canadian ROKband Pediatric Headshape Clinics in 2023. CROs were fabricated according to standard clinical guidelines, which are based on a topical scan of the infant’s cranial shape. The scan is then modified by the manufacturer using custom software (AutoCoco, New Westminster, BC, Canada) to create void space where the cranial shape is flattened and to touch the areas where the cranial shape is bossed. This modified shape varies based on the infant’s cranial measurements at the time of initiation of orthotic treatment and are based on the clinical goals of treatment (which is typically normalization of the cranial shape based on desired post-treatment measurements). This scan modification process mirrors fabrication methods of traditionally fabricated CROs.

**Figure 1:**
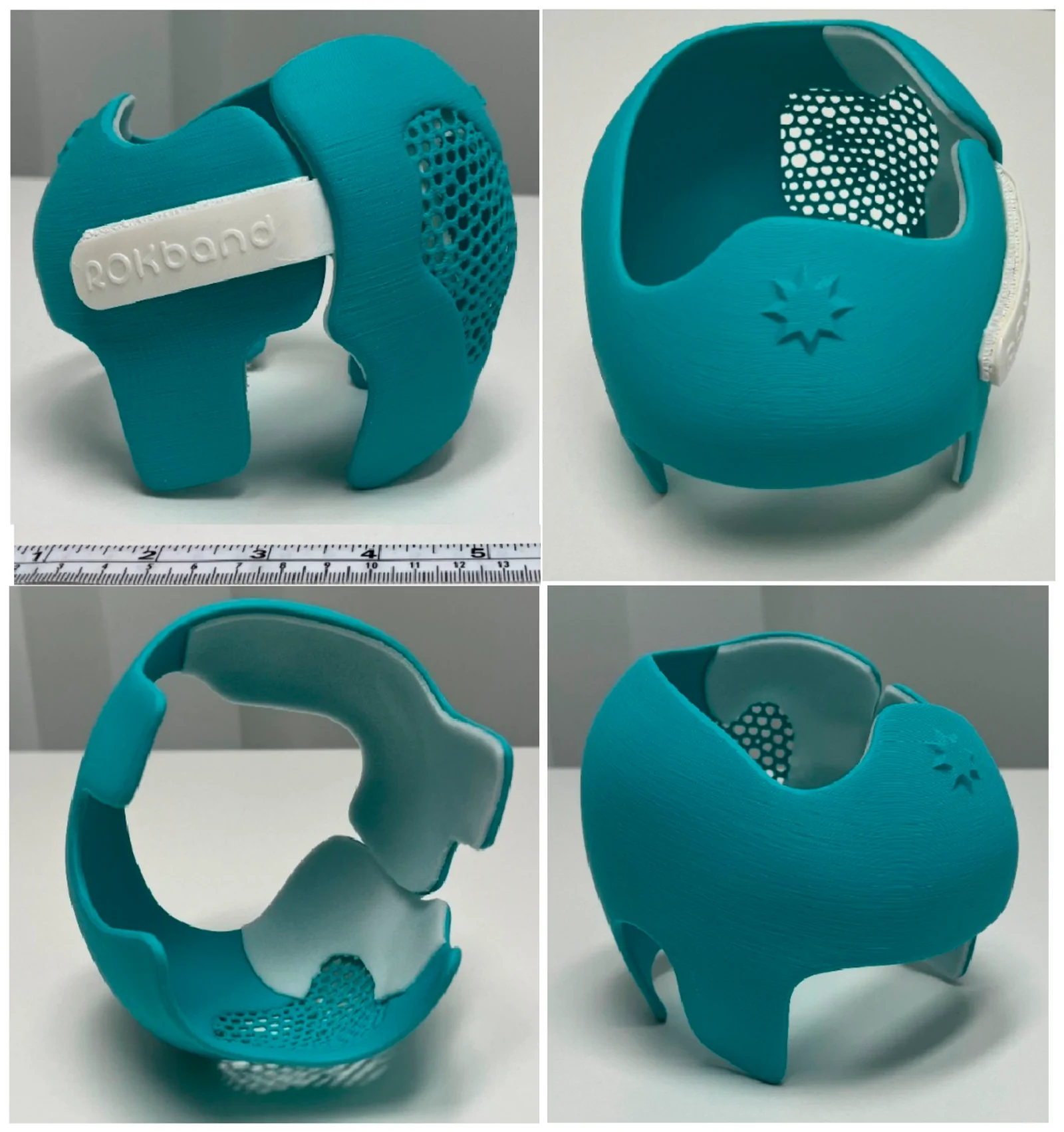
Example photos of a custom-fabricated ROKband Cranial Remolding Orthosis.

Uniquely, the ROKband treatment program includes up to two custom-made 3D-printed CROs for each participant (Figure 1), which are 3D-printed in plastic and an orthopedic-grade lining is added after printing. In cases where two CROs are needed during treatment, the second CRO is provided at no additional cost to the family.

Collected data for the study included the age at initiation of CRO treatment, presenting and post-treatment head shape measurements as calculated by Cranial Vault Asymmetry Index (CVAI) and Cephalic Ratio (CR), duration of treatment, and parental satisfaction with treatment. Participants were separated by head shape type and treatment initiation age. In the cases where the participant was born prior to 38 weeks gestation, treatment initiation age was corrected based on the infant’s gestational age (treatment initiation age equals the infants chronological age minus the number of weeks of prematurity).

### 2.1. Inclusion/Exclusion Criteria

Participants were included if they had a diagnosis of DP, DB or DAB. Infant’s corrected ages ranged from four to 12 months at the initiation of treatment. Data was stratified by age group and head shape diagnosis. The eligible participants wore the CRO 20–23 h/day. Infants were excluded if they were diagnosed with dolichocephaly/scaphocephaly (longer than typical head shapes) or asymmetric dolichocephaly/scaphocephaly (head shapes that are long and asymmetric), had complex medical needs that could affect their cranial growth (such as genetic syndromes), or had non-compliance with treatment (as reported by the caregivers or treating practitioner). Since wear time can only be reported subjectively, criteria for non-compliance included reviewing the practitioner’s notes at each scheduled follow-up visit (typically every 3–4 weeks) to determine if there had been inconsistent wear time reported by the caregivers during more than two months of the treatment, if families had greater than two months between follow-ups, and/or repeated failure to attend regularly scheduled follow-up appointments. This corresponds to standard clinical practice regarding the utilization of caregiver-reported wear times in this population [22,26,28,34].

### 2.2. Procedures

**Figure 2:**
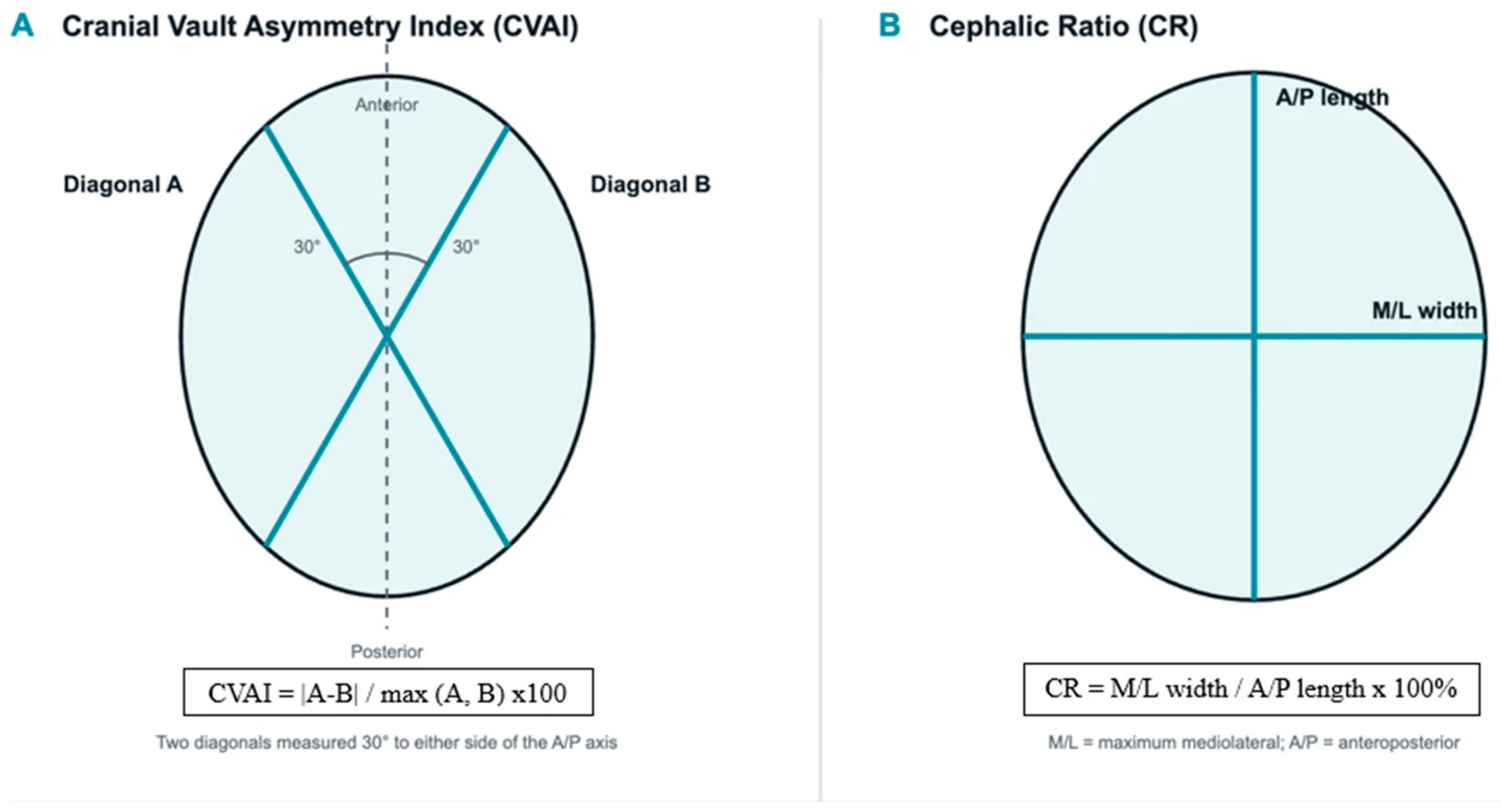
Calculations for Cranial Vault Asymmetry Index and Cephalic Ratio, which are measured at the greater equator of the skull.

All assessments were completed using a Peel 3D light scanner (Creaform, New Westminster, BC, Canada) [37] and CRO adjustments were performed every 3–4 weeks. Scans were completed at every follow-up and compared with the client’s previous scan and measured using CraniAlign (New Westminster, BC, Canada) [38]. These scans were conducted by the same 2–3 clinicians at the client’s respective clinic locations, with a few exceptions if the families were travelling to other provinces. The measurements from the scans were used to calculate the CVAI and CR of each infant (Figure 2). CVAI measurements were based on the Children’s Healthcare of Atlanta (CHOA) guidelines, which use the longer cranial diagonal as the denominator [39]. Although there is no official standardized classification of the normal CR, correction criteria were based on previously studied norms [40].

To minimize measurement variability, scans were obtained using standardized infant positioning, scanning-cap placement, and CraniAlign processing procedures. Scanners were calibrated two to three times annually per manufacturer protocols, and scans were repeated when scans included movement waves or artifacts, or when clinicians determined that a rescan was needed for clinical accuracy.

Clinician notes were analyzed to interpret treatment adherence and reason for discharge. The fitting of the CRO was 5–14 days after the initial scan. The ‘final/graduation’ scan was taken within four weeks of discontinuation of treatment. Discharge criteria were based on numerous factors, including improvement of head shape into normal limits, outgrowth of CRO(s), familial satisfaction with head shape, and caregiver choice. Normal limits were defined as a CVAI < 3.5% and a CR ≤ 90%, and infants were considered to be “successful” with CRO treatment if their head shape improved to below this threshold.

Caregiver satisfaction was measured through analysis of clinician notes, caregiver’s graduation surveys and emails between families and clinicians. Clinician notes had to specify that the ‘family/caregiver was happy with care and results’ to be considered satisfied. Satisfaction was separated into three categories: “Happy with results”, “Unsure/unclear how they felt about results”, and “Not happy with results”.

### 2.3. Data Analysis Methods

Participants were organized into groups based on the adjusted age of initiation of CRO treatment (<6 months, 6–9 months, and 9–12 months) and head shape category (DP, DB, and DAB). Initial cranial measurements (CVAI and CR) were reported based on the CRO fabrication scan and the post-treatment measurements were based upon the scan at the discontinuation of treatment. Participant information was de-identified and stored in a shared electronic file according to the Data Use Agreement between Pediatric Headshape Clinic and the University of Texas Southwestern Medical Center. Descriptive statistics were used to summarize demographic data and measurements including total number in each category, cranial measurements, range, standards of deviation, and gender. Two-sample *t*-tests or Mann–Whitney U tests were performed to compare measurements between head shape groups when appropriate after checking normality assumption using Shapiro–Wilk tests. Fisher’s exact tests were applied to compare success and satisfaction rates. In addition, logistic regression models were conducted to investigate the association between success rate with adjusted age of initiation of CRO treatment, treatment duration, and continuous initial CVAI/CR measurements. Statistical analyses were conducted using SAS 9.4 (SAS Institute Inc., Cary, NC, USA) and the significance level was set to 0.05.

### 2.4. Ethics Statement

IRB approval for this study was obtained through the University of Texas Southwestern Medical Center’s institutional review board (Study #STU-2025-0391). Due to the retrospective design of the study, no direct recruitment of subjects occurred. Any clinically-related recruitment or consenting was done by Pediatric Headshape Clinic prior to study, in that the subjects agreed to receive treatment at one of their clinics. A waiver of consent and data use agreement was utilized to collect data from the involved clinics for infants diagnosed with DP, DB, or DAB and treated with a CRO. Data was securely transferred to UTSW for analysis via a data use agreement (Contract #DUA202311-0008).

## 3. Results

In total, 1054 infants were treated in 2023 at the participating clinics who met the inclusion criteria for this study. Across all head shapes, the youngest treatment initiation group (<6 months) had the most participants (DP n = 278, DB n = 67, and DAB n = 217). For infants aged 6–9 months, there were 224 DP participants, 64 DB participants and 148 DAB participants. For 9–12 months, there were 25 DP participants, eight DB participants and 23 DAB participants (Table 1).

**Figure 3:**
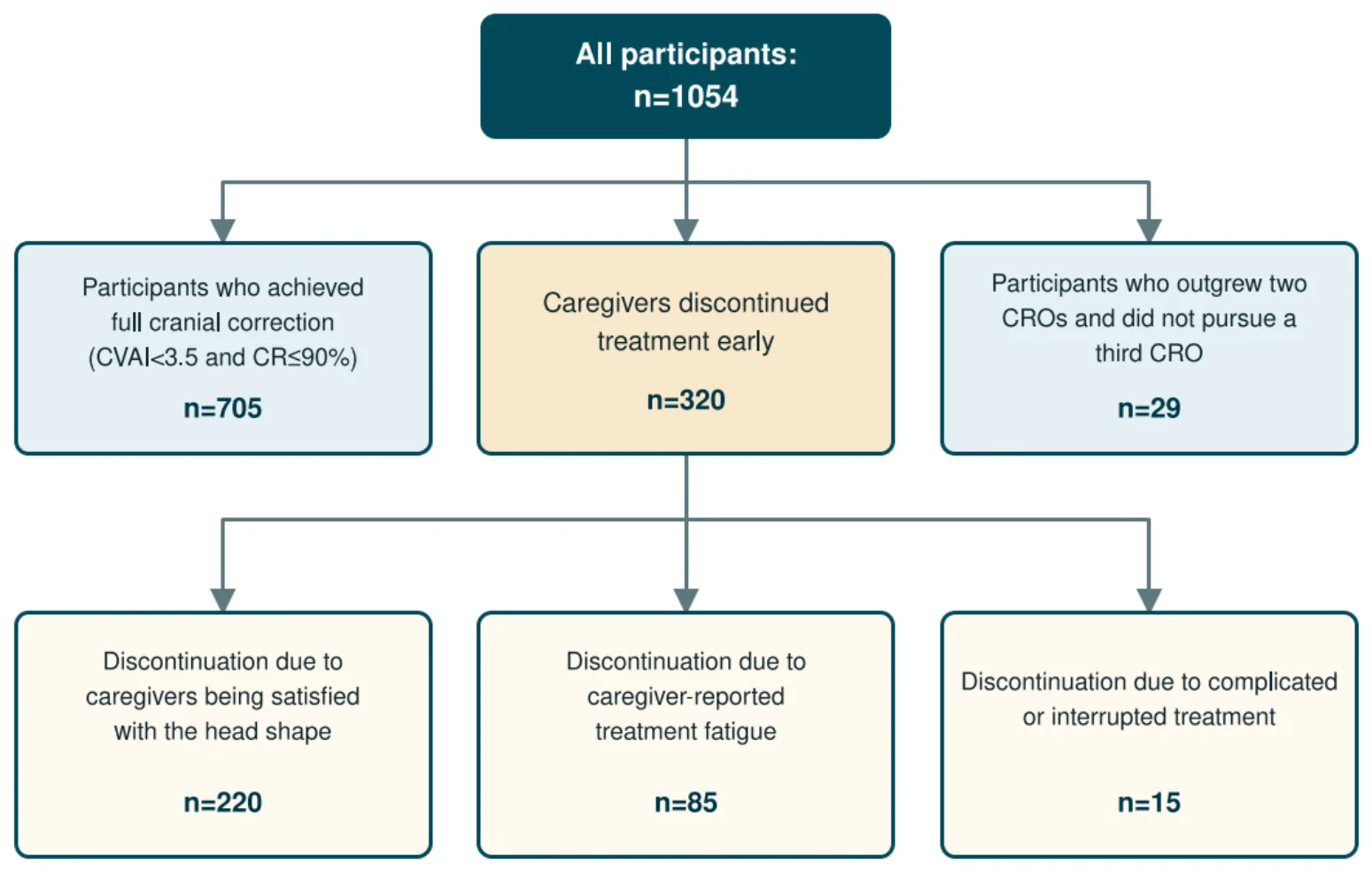
Flow chart detailing the reason for discontinuation of CRO treatment in the study cohort.

The majority of the infants (705) achieved full cranial correction as defined by our study criteria. Of the 1054 participants, 782 (74.2%) required a second orthosis to achieve their final treatment outcome. A total of 29 infants outgrew both CROs provided in the standard clinical care of the participating offices and chose not to pursue a third CRO. Additionally, 320 infants discontinued treatment before the clinician recommended CRO discontinuation, for which reasons did vary. A total of 220 of these infants discontinued treatment because the caregiver was satisfied with the treatment outcome. Furthermore, 85 infants discontinued treatment because the caregiver reported that they were fatigued with the treatment process, cleaning the CRO, or the required follow-up visits that are necessary to adjust the CRO for cranial growth. A total of 15 of the 1054 infants had complications with CRO use or a life situation that required the discontinuation of the CRO. Figure 3 visually depicts the reasons for discontinuation of CRO treatment in this cohort. Examples of a complicated or interrupted treatment are: 1) extended trips where the family did not wish to wear the CRO, 2) increased temperature or heat waves that discouraged families from continuing, 3) patient beginning daycare or schooling and did not wish to wear the CRO and 4) sicknesses or illnesses that led to long durations of not wearing the CRO, which ultimately led to them stopping treatment.

### 3.1. Overall Success Rates

Successful treatment was defined as full correction of the head shape into the mildest CHOA category (CVAI < 3.5) [39] and a CR ≤ 90%. Overall, 66.89% (705/1054) of the participants had successful treatment of their cranial shape but this did vary by age of treatment initiation and head shape category (Table 2). For example, success rates ranged from 82.73% in the youngest DP group to 21.74% in the oldest DAB group.

### 3.2. Success Rate Stratified by Initial Age and Head Shape

Table 2 demonstrates that across all head shapes, the overall success rate of the <6 months participants was highest (72.95%), followed by the 6–9 months group (62.61%), then the 9–12 months group (39.29%). The DP head shape had the highest overall correction rates, with 82.73% success for the <6 months group, 71.88% correction for the 6–9 months group, and 56.00% for the 9–12 months group. The DAB head shape had the next highest overall success rates (64.06% success for <6 months group and 54.05% for 6–9 months group). The 9–12 months DAB participants had the lowest success rate in the study, with only 21.74% achieving full correction to both their CVAI and CR measurements. The DB head shape had the lowest number of participants as well as the lowest correction rates when evaluating all ages combined (54.68% overall CR correction rate).

Based on Fisher’s exact test, CRO treatment initiation age has a significant impact on success rates for participants with DP (*p* = 0.0007) and DAB (*p* = 0.0003). Initiation age for DB was not found to have a statistically significant impact on treatment success (*p* = 0.2744).

Based on logistic regression analysis, both CRO treatment initiation age and initial cranial measurements (CVAI for DP, CR for DB, CVAI and CR for DAB) have significantly negative associations with success rates for participants with DP, DB, and DAB (all *p* < 0.0001) after adjusting for treatment duration. In addition, the treatment duration is not significantly associated with the success rate after adjusting for initiation age and initial cranial measurements for three head shapes. The odds ratios and specific *p*-values of this logistic regression model are presented in Table 3.

### 3.3. Head Shape Measurement Change

#### 3.3.1. Deformational Plagiocephaly (DP)

The overall CVAI improvement for all DP head shapes was 5.07 (Table 4). In general, the younger the infant was at the initiation of treatment, the higher the change in CVAI. In the <6 months age group, the mean CVAI improvement was 5.73 (range: 1.89 to 16.51); in the 6–9 month DP group, the mean improvement was to 4.41 (range: 1.3 to 10) and in the 9–12 month group, the mean change was 3.69. The range of correction also decreased in this older age group (range: 1.41 to 6.66).

#### 3.3.2. Deformational Brachycephaly (DB)

The overall CR improvement across all DB participants was 6%. The mean CR improvement for each age group was 7% (<6 months, range 2 to 13%), 5% (6–9 months, range 1 to 15%), and 4% (9–12 months, range 2 to 7%).

#### 3.3.3. Deformational Asymmetrical Brachycephaly (DAB)

For DAB head shapes, the overall CVAI improvement was 4.79 and CR was 5%. The <6 months group had a mean change of 5.29 for CVAI (range: 1.35 to 11.10) and 5% for CR (range: 1% to 13%), whereas in the 6–9 months age group, there was a mean improvement of 4.28 for CVAI (range: 0.98 to 11.03) and 4% for CR (range: 0 to 8%). In the 9–12 month age group, the mean change for CVAI was 3.32 (range: 1.31 to 7.27) and CR was 4% (range: 1% to 7%).

#### 3.3.4. Head Shape Group Comparisons

DB participants had greater improvement in CR compared to DAB in the <6 months group (7% vs. 5%) and 6–9 months group (5% vs. 4%), which was statistically significant (*p* < 0.0001 and *p* = 0.0004, respectively).

DP participants had significant improvements in CVAI compared to DAB in the <6 months group (5.73 vs. 5.29, *p* = 0.0205). There was no statistically significant difference in the improvement of CVAI when comparing the DP or DAB groups in the 6–9 months group (4.41 vs. 4.28) or 9–12 months age group (3.69 vs. 3.32) with *p* = 0.5638 and *p* = 0.1732, respectively. However, head shapes did improve in both of these categories.

### 3.4. Caregiver Satisfaction

Caregiver satisfaction remained high across all head shapes and age groups (Table 5). Overall, 995/1054 (94.40%) caregivers were satisfied with their child’s post-treatment head shape. When looking at the treatment initiation age, caregiver satisfaction in the <6 months and 6–9 months was highest (96.26% and 93.58%, respectively). In the 9–12 months group, the caregiver satisfaction rate was 82.14%. When evaluating the different head shapes (across all age groups), DP parental satisfaction was 93.36%, DAB was 95.10% and DB was 96.40%. Although the overall parental satisfaction was high, DP and DAB had a lower satisfaction rate in the older age groups (9–12 months) compared to other age groups, which was statistically significant (*p* = 0.0325 and *p* = 0.0046, respectively). Due to the limited number of participants in the older age group with DB, additional data would be needed to examine the relationship between age groups. Among the caregivers who did not explicitly express their satisfaction with the CRO treatment to their providing clinician, there were also no reports of the caregivers being dissatisfied and therefore these satisfaction ratings were categorized as “unsure/unclear how they felt about results”.

## 4. Discussion

### 4.1. Success Rate with 3D-Printed CRO and Head Shape Type

3D-printed devices are becoming more popular in the orthotics community, as they provide a lightweight, more comfortable option for patients [41]. While most existing literature reports high success rates with traditionally fabricated CROs [22,30,31,42,43], evidence regarding 3D-printed CROs remains limited. Recently, a study completed by Willenborg et al. assessed the effectiveness of 3D-printed CROs and parental satisfaction [36]. Their study concluded that 3D-printed CROs were effective at correcting DP, DAB and DB head shapes and that age, severity and head shape type had an impact on the success rate [36]. Our research findings were also able to show that treatment success varied by deformation type and CRO initiation age for all three observed head shapes: DP, DAB, and DB.

Of the 1054 participants, post-treatment cranial measurements demonstrated that infants with DP have the highest treatment success (76.85%), followed by DAB (57.73%), and the lowest success rate was seen for DB (54.68%). This finding is aligned with previous research that indicates DB head shapes have lower rates of full correction in traditionally fabricated CROs compared to DP [44]. Studies examining CRO outcomes define success differently. While our study used CVAI < 3.5% and CR ≤ 90% as the marker for success, other studies have used ODD ≤ 5 mm [22,45], CVAI ≤ 7 [46], and other definitions such as improving head shape by at least one severity category [36]. This distinction is important as clinician notes indicated caregivers often chose to self-discharge from the ROKband program once milder measurements were achieved, largely due to satisfaction with the treatment. This was particularly noticeable when analyzing the DB and DAB categories. Although these infants corrected into mild categories, our findings would have considered their treatment unsuccessful if they did not measure CVAI < 3.5% and CR ≤ 90%.

While the current literature frequently references the CHOA scale for success of asymmetry (CVAI < 3.5) [39], there is limited literature regarding standards for success regarding DB head shapes [47,48]. In this study, we used the CR ≤ 90% as the threshold for successful correction of the CR because this was the standard used at the treating clinics and has been used in other studies [24,40,42,43]. If this threshold was changed to CR ≤ 91%, the number of DB infants who would be reported as achieving full correction of their CR would dramatically increase from 76/139 (54.68%) to 99/139 (71.22%) and the DAB correction rate would have changed from 224/388 (57.73%) to 267/388 (68.81%). This highlights the potential need to investigate the current literature standards for normal head shapes and the need to take into consideration where familial satisfaction lies with these numbers.

On average, participants with asymmetric head shapes were able to improve their CVAI by 4.95 and brachycephalic head shapes were able to improve their CRs by 5%. The lowest CR change for some subjects was 0% and found in the DAB group. While this may suggest that some participants did not get any correction, there was still a CVAI reduction for these infants; therefore, the CRO did still improve the cranial shape. Another point to consider when evaluating this study is that this treatment program included up to two CROs, if required. Void space in each 3D-printed CRO was determined by the patient’s age, severity and expected growth rate. In one rare case, a participant outgrew both of the CROs included in their treatment program and purchased a third. Though this is uncommon, it highlights the possibility that other participants who may not have fully corrected their head shape opted not to purchase a third CRO, potentially limiting the ability to get full correction of the head shape.

### 4.2. Age and Success Rate

There is a vast amount of literature that supports early initiation of traditionally-fabricated CROs for more successful outcomes, with most studies recommending to begin treatment before 6 months of age [22,25,27,28,49,50,51,52]. Similarly, our findings show that the highest success rate using 3D-printed CROs was seen in the youngest patients (<6 months) with a rate of 72.95% across all head shape types while the oldest group (9–12 months) had a success rate of 39.29%. When comparing disproportionate head shapes, it was noted that the 9–12 months group had greater success in DB than DAB, whereas the two younger age groups had greater success in DAB than DB. The DP group had the highest success rates within every age group. These results suggest age has a strong impact on the treatment of asymmetric head shapes (DP and DAB), but may not be as strong a predictor of success in DB. One possible explanation is the natural improvement observed in some cases of brachycephaly with increasing age and greater time spent in the prone position [53,54]. Additional studies have indicated repositioning efforts for asymmetric head shapes can be effective prior to six months of age, suggesting natural head shape improvement for DP and DAB is unlikely to occur in older age groups [8,55]. This hypothesis would support our findings, as DB may improve naturally as children get older.

In recent years, the increasing CR of the general population has been attributed to the strong promotion of supine-sleeping [47,48]. As many babies’ heads are now wider [48], caregivers are more accustomed to brachycephalic head shapes. Race and sex can also play a role in normative values of CR [56,57]. In 2010, a study concluded that people of Chinese descent had rounder and wider head shapes than Caucasians [58]. Rogers (2010) describes babies that are born male as having larger and wider head shapes due to faster growth [59]. These genetic factors may impact a caregiver’s choice to continue CRO treatment once the infant gets into mild ranges. Additional research is needed to get a better understanding of the role that race and sex have on head shape [47].

### 4.3. Caregiver Satisfaction

The clinical protocol of the participating clinics included a caregiver exit survey upon graduation from 3D-printed CRO treatment. When reviewing these surveys, caregiver satisfaction was consistently high, ranging from 80% in the 9–12 months DP group to 97.7% in the <6 months DAB group. Our results suggest a negative correlation between age and caregiver satisfaction: as treatment initiation age increases, caregiver satisfaction decreases. Prior research shows that initiating CRO treatment at a younger age results in a shorter treatment duration [50,51,60,61], greater head shape correction [3], and better clinical outcomes [25,61], which may explain why the caregivers in this study reported higher treatment satisfaction when treatment was initiated at a younger age.

In comparison to younger age groups, the 9–12 months group had lower caregiver satisfaction across all head shapes. It could be inferred that due to lower success rates in the older age group, caregivers may have been slightly less satisfied with the results. However, there was no significant difference between the caregiver satisfaction rate in DP, DB or DAB across all age groups (*p* = 0.3216). This suggests regardless of an infant’s head shape type at the initiation of treatment, caregivers were typically satisfied with the treatment outcome.

### 4.4. Limitations

This study was a retrospective cohort study, which limited researcher control over data collection. Moreover, interpretation and judgement between clinicians could have influenced scan alignment and clinical decisions during the course of treatment [62]. As there were minor variances between clinician notes, there may have been some interpretation bias within the researchers who were reading the notes.

Compliance was primarily assessed through caregiver reports, introducing the potential for reporting bias. There also may have been a potential selection bias, as only patients that were considered ‘compliant’ with treatment were included in the analysis. This may have excluded families who may have experienced adverse effects that did not allow them to be fully compliant with treatment. Objective wear-time monitoring with a wear-time sensor is not yet standard clinical practice for cranial remolding [28,63,64], and as such, there were no objective measurements of CRO wear time able to be collected in this study. It is possible that caregivers overrepresented the amount of time the CRO was actually being worn, which has been found to be the case in two existing studies that objectively evaluated CRO wear-time [63,64].

A formal assessment of inter-rater and intra-rater reliability was not conducted for the Peel 3D–CraniAlign measurement process in this study, which may have introduced measurement variability across clinicians and sites. The Peel 3D scanner has a manufacturer-reported accuracy of up to 0.250 mm and measurement resolution of 0.250 mm [65]. Standardized 3D craniofacial measurements and acquisition protocols have demonstrated good-to-excellent reliability and reduced measurement error in previous studies [66,67,68].

Another limitation of this study is that the treatment program that was completed by the participants of this study included up to two CROs, if required. Other treatment programs that used 3D CRO devices may only include one device, which would require either purchasing a second CRO to achieve full correction, or potentially result in lower success rates. One participant in the observed cohort outgrew both of the CRO’s included in their treatment program and ended up purchasing a third. Though this is rare, it shines light on the possibility that the 2.75% (29/1054) of participants who opted not to purchase a third CRO may have still been able to achieve cranial correction if they had elected to continue treatment.

Three of the study authors are currently employed by ROKband Pediatric Headshape Clinics, which are owned by the company providing support for this study and the manufacturers of the ROKband CRO (Headstart Medical); however, the authors at the University of Texas Southwestern Medical Center were included in order to limit the potential for bias and to independently analyze and interpret the results of the study. All subjects who met the study-defined inclusion criteria were included and the concept of this study had not yet been conceived at the time the patients were treated. All infants were treated by active certified clinicians at Pediatric Headshape Clinic in 2023, and discontinued CRO use before data collection began. Scan measurements were recorded by the treating clinicians during the course of active treatment for the cranial shape.

Due to the retrospective nature of this study, the results represent a limited cohort of infants treated with the ROKband brand 3D-printed CRO in Canada, and may not be generalizable to the broader population treated with the same or different brand of 3D-printed CRO due to each clinic potentially having their own respective clinical protocols for follow-up visits, documentation, and CRO wear-time.

This study did not include a control group of untreated infants for a comparison of ROKband treatment to the natural course of cranial growth nor did it include a comparison group of infants treated with traditionally fabricated CROs.

Table 1 demonstrates that the participants were not evenly distributed among treatment age and deformation subcategories, as the vast majority of participants were in the younger age groups. Only 5.31% of the studied population were in the 9–12 month treatment initiation age group. Though this is consistent with evidence surrounding the importance of starting CRO therapy at a younger age, which has informed clinical practice to recommend treatment initiation at younger ages, this may have affected the statistical strength of the results for this older age group. DB was also likely underrepresented for definitive statistical comparisons as this is the least common of the three studied cranial deformation types (13.19% of the observed cohort). Five age–deformation–gender subgroups had fewer than 15 infants, so genders were combined for analysis. Combining genders in the observed cohort subgroups resulted in only one group with fewer than 15 participants (the 9–12 month DB group), which included only eight participants. In a recent large retrospective cohort study (27,990 subjects) evaluating factors that are associated with the efficacy of traditionally fabricated CROs, biological sex was not found to be a significant predictor of CVAI correction [28]. This is consistent with a study published one year earlier that found that biological sex had no significant impact on cranial asymmetry correction [30]; therefore, biological sex was not used as a stratification variable in the present analysis.

## 5. Conclusions

The treatment protocols at the six observed Canadian clinics utilizing 3D-printed CROs resulted in a reduction in the cranial deformation in all participants. A greater number of infants achieved full correction of their cranial shape when treatment was initiated at a younger age, which supports many existing studies that have correlated more optimal treatment outcomes with earlier CRO intervention [26,27,28,42,50,51,61]. Although the caregiver satisfaction rate remained high across all participants, it was the highest amongst the youngest age group (<6 months). These results add to the body of knowledge surrounding treatment for deformational head shapes and the limited evidence surrounding the efficacy of 3D-printed CROs [36]. These results may help provide valuable insights to support families considering CRO treatment and practitioners who are attempting to determine the optimal timing for CRO intervention.

## Figures and Tables

**Table 1:** Participant demographics based on initial age at initiation of CRO treatment.

Pre-Treatment Participant Age and Head Shape Distribution				
Age Group	DP	DB	DAB	Total
<6 months (all)	278	67	217	562
<6 months (male)	175	44	155	374
<6 months (female)	103	23	62	188
6–9 months (all)	224	64	148	436
6–9 months (male)	154	38	103	295
6–9 months (female)	70	26	45	141
9–12 months (all)	25	8	23	56
9–12 months (male)	14	6	15	35
9–12 months (female)	11	2	8	21
Total	527	139	388	1054

**Table 2:** Success rates based on initial age and head shape (final CVAI < 3.5 and CR ≤ 90%).

Participants Who Achieved Full Cranial Correction Based on Age and Head Shape Subgroups				
Age Group	DP	DB	DAB	Total
<6 months	230 (82.73%)	41 (61.19%)	139 (64.06%)	410 (72.95%)
6–9 months	161 (71.88%)	32 (50.00%)	80 (54.05%)	273 (62.61%)
9–12 months	14 (56.00%)	3 (37.50%)	5 (21.74%)	22(39.29%)
Total	405 (76.85%)	76 (54.68%)	224 (57.73%)	705 (66.89%)

**Table 3:** Results of the logistic regression models showing the associations between measured variables and the treatment success rate (final CVAI < 3.5 and CR ≤ 90%).

Logistic Regression Model			
Factor	DP Odds Ratio (95% CI) *p*-value	DB Odds Ratio (95% CI) *p*-value	DAB Odds Ratio (95% CI) *p*-value
Initial Age	0.98 (0.98, 0.99) <0.0001 *	0.97 (0.96, 0.99) <0.0001 *	0.98 (0.97, 0.99) <0.0001 *
Treatment Duration	1.00 (0.99, 1.00) 0.4322	1.00 (0.99, 1.01) 0.8854	1.00 (0.99, 1.01) 0.1101
Initial CVAI	0.55 (0.48, 0.63) <0.0001 *	n/a	0.65 (0.56, 0.75) <0.0001 *
Initial CR	n/a	<0.01 (<0.01, <0.1) <0.0001 *	<0.01 (<0.01, <0.1) <0.0001 *

[* indicates statistical significance]. [“n/a” indicates this measure is not applicable to the head shape].

**Table 4:** Participant demographic description, means and ranges.

Participant Pre-Treatment Demographics					
Age Group	Cranial Measurement (Mean ± SD)		DP	DB	DAB
<6 Months	CVAI	Initial Measurement	8.42 ± 2.41	n/a	7.45 ± 2.24
		Decrease	5.73 ± 2.05	n/a	5.29 ± 1.96
		Range of Decrease	1.89–16.51	n/a	1.35–11.1
	CR	Initial Measurement	n/a	98% ± 4%	95% ± 3%
		Decrease	n/a	7% ± 3%	5% ± 2%
		Range of Decrease	n/a	2–13%	1–13%
6–9 Months	CVAI	Initial Measurement	7.47 ± 2.07	n/a	6.95 ± 2.16
		Decrease	4.41 ± 1.64	n/a	4.28 ± 1.72
		Range of Decrease	1.30–10.00	n/a	0.98–11.03
	CR	Initial Measurement	n/a	96% ± 3%	94% ± 3%
		Decrease	n/a	5% ± 2%	4% ± 2%
		Range of Decrease	n/a	1–15%	0–8%
9–12 Months	CVAI	Initial Measurement	7.59 ± 2.13	n/a	6.81 ± 2.00
		Decrease	3.69 ± 1.28	n/a	3.32 ± 1.41
		Range of Decrease	1.41–6.66	n/a	1.31–7.27
	CR	Initial Measurement	n/a	96% ± 5%	94% ± 3%
		Decrease	n/a	4% ± 2%	4% ± 2%
		Range of Decrease	n/a	2–7%	1–7%
All Ages Combined	CVAI	Initial Measurement	7.98 ± 2.30	n/a	7.22 ± 2.21
		Decrease	5.07 ± 1.98	n/a	4.79 ± 1.94
		Range of Decrease	1.30–16.51	n/a	0.98–11.10
	CR	Initial Measurement	n/a	97% ± 4%	95% ± 3%
		Decrease	n/a	6% ± 3%	5% ± 2%
		Range of Decrease	n/a	1–15%	0–13%

[“n/a” indicates this measure is not applicable to the head shape].

**Table 5:** Caregiver satisfaction rates based on initial age and head shape.

Caregiver Satisfaction Ratings				
Age Group	DP	DB	DAB	Total
<6 months	263 (94.60%)	66 (98.51%)	212 (97.70%)	541 (96.26%)
6–9 months	209 (93.30%)	61 (95.31%)	138 (93.24%)	408 (93.58%)
9–12 months	20 (80.00%)	7 (87.50%)	19 (82.61%)	46 (82.14%)
Total	492 (93.36%)	134 (96.40%)	369 (95.10%)	995 (94.40%)

## Data Availability

The University of Texas Southwestern Medical center requires participants to be notified in the informed consent form if a participant’s individual data will be shared through a controlled or open access repository and the risks and benefits associated with each sharing method. When this study was performed, a waiver of consent was obtained and therefore we cannot publicly share data at the participant level. Data may be requested through a data use agreement by contacting tiffany.graham@utsouthwestern.edu.

## Abbreviations

The following abbreviations are used in this manuscript:

CVAI	Cranial Vault Asymmetry Index
CR	Cranial Ratio
DB	Deformational Brachycephaly
DAB	Deformational Asymmetric Brachycephaly
DP	Deformational Plagiocephaly
CHOA	Children’s Healthcare of Atlanta
